# Qualitative and Quantitative Analysis of Tumor Cell Invasion Using Au Clusters

**DOI:** 10.3390/nano12010145

**Published:** 2021-12-31

**Authors:** Xiangchun Zhang, Qinqin Zheng, Ziqi Wang, Chao Xu, Haolei Han, Aiping Li, Guicen Ma, Jiaojiao Li, Chengyin Lu, Hongping Chen, Zhichao Zhang

**Affiliations:** 1 Tea Research Institute, Chinese Academy of Agricultural Sciences, Hangzhou 310008, China; zhangxc@tricaas.com (X.Z.); zhengqinqin@tricaas.com (Q.Z.); wangziqi199710@163.com (Z.W.); 18482004388@163.com (H.H.); AdelapLive@outlook.com (A.L.); mgc1314@tricaas.com (G.M.); lchy@tricaas.com (C.L.); 2 College of Chemistry and Material Science, Shandong Agricultural University, Taian 271018, China; xuc@sdau.edu.cn; 3 Department of Chemistry and Biology, Faculty of Environment and Life Science, Beijing University of Technology, Beijing 100124, China; lijiaojiao@emails.bjut.edu.cn; 4 Department of Musculoskeletal Tumor, Fudan University Shanghai Cancer Center, Shanghai 200032, China

**Keywords:** Au clusters, MT1-MMP, invasion/metastasis, human cervical cancer

## Abstract

Tumor invasion/metastasis is still the major cause of death in cancer patients. Membrane type-1 matrix metalloproteinase (MT1-MMP) is directly related to tumor invasion/metastasis. To accurately and quickly distinguish the risk of invasion/metastasis of primary tumor cells, it is urgent to develop a simple and precise quantitative method to distinguish the expression level of MT1-MMP. In this work, we have constructed red fluorescent Au clusters with peroxidase-like properties that could specifically bind to MT1-MMP on human cervical cancer cells. After MT1-MMP was labelled with Au clusters, we could visually see red fluorescence of MT1-MMP on cervical cancer cells via fluorescence microscopy and catalytic color imaging using an ordinary optical microscope. The constructed Au clusters contained 26 Au atoms; thus, the amount of MT1-MMP on cervical cancer cells could be accurately quantified using inductively coupled plasma mass spectrometry (ICP-MS). More importantly, the invasion/metastasis capabilities of the cervical cancer Siha, Caski and Hela cells with different MT1-MMP amounts could be accurately distinguished by fluorescence/catalysis qualitative imaging and ICP-MS quantitative analysis. This method of qualitative/quantitative analysis of tumor-associated proteins on cancer cells has great potential for accurately diagnosing aggressive tumor cells and assessment of their invasion/metastasis risk.

## 1. Introduction

Despite the tremendous progress in understanding the occurrence of cancer, the 5-year survival rate of distant metastases tumors has not improved significantly compared to the localized tumor in most cancer patients, and invasion/metastasis is still the main cause of death in cancer patients [1,2,3,4]. The occurrence and development of tumors are accompanied by the abnormal expression of multiple biomarkers. Before tumor metastasis, tumor cells must invade the extracellular matrix (ECM) of the primary tumor [5,6,7]. In the invasion process, matrix metalloproteinases (MMPs) play a deterministic role in tumor cells invading the surrounding microenvironments of cleaving the ECM [8,9,10], and MMPs are closely related to cancer cell growth, invasion, angiogenesis, and metastasis [11,12,13,14]. Among many kinds of MMPs, membrane type-1 matrix metalloproteinase (MT1-MMP) is extremely important, as it directly degrades several ECM molecules, such as collagen types I, II, III, laminins-1 and -5, fibrin, fibronectin, vitronectin, and aggrecan [15,16,17,18,19,20]. On the other hand, MT1-MMP has the capacity to degrade the ECM indirectly via activating pro-MMP-2 on cells and promoting angiogenesis [15,21]. Moreover, MT1-MMP was used as a potential targeting biomarker for tumor diagnosis and prognosis, including lung cancer, renal cancer, cervical cancer, glioblastomas, prostate cancer and breast cancer [18,22,23,24,25]. Therefore, there is an urgent need to develop a multi-functional integrated protein analysis method to monitor MT1-MMP.

Thus far, the commercial protein quantification and analysis methods are mainly based on the principle of antibodies, including enzyme-linked immunosorbent assay (ELISA), immunoblotting, immunohistochemistry (IHC) and immunofluorescence (IF) [26,27,28]. Unfortunately, ELISA and immunoblotting require a large number of cell population lysis and protein extraction, which is cumbersome, and protein loss is inevitable during the processing and cannot offer enough sensitivity for certain proteins [26]. IF and IHC can detect proteins on intact cells at the single-cell level, but the sampling process is time-consuming and the fluorescence under multi-component biological samples is easy to quench. To tackle this issue, inductively coupled plasma mass spectrometry (ICP-MS) has been employed in the biological field to detect metal-labelled biological proteins due to its high sensitivity and accuracy [29,30,31]. In recent years, metal clusters with ultra-small size, unique fluorescent properties, precise molecular formulas, and exhibit better good biocompatibility that has been attractive to the analytical chemist [32,33,34,35,36,37,38]. In 2014, Liu et al. constructed a silver cluster containing three silver atoms that could label the immunoglobulin M (IgM) on the surface of Ramos cell membrane with a green fluorescent, and the IgM could be quantified by ICP-MS [29]. Subsequently, the Au_24_ clusters with red fluorescent were reported to label and quantify the cell membrane protein integrins α_IIb_β_3_ in HEL cells in situ [30]. These methods can be performed in situ quantitative analysis of tumor cell protein on single cells, but whether these metal cluster probes can assess the invasion/metastasis ability of tumor cells with different degrees of differentiation has not been reported.

**Scheme 1:**
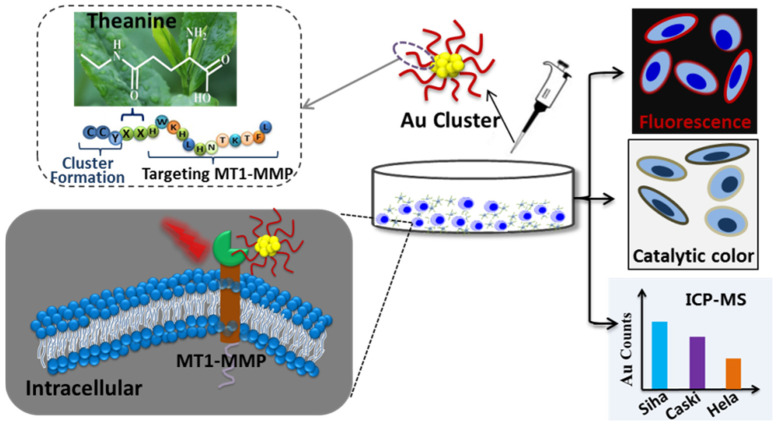
Schematic illustration of the qualitative and quantitative distinction of human cervical cancer cells with different invasion/metastasis capabilities by using Au clusters.

Here, we constructed a multifunctional Au clusters probe using artificially designed peptides. The constructed Au clusters had inherent red fluorescent properties and peroxidase-like properties. It could catalyze the oxidation of 3,3′-diaminobenzidine tetrahydrochloride (DAB) and 3,3′,5,5′-tetramethylbenzidine sulfate (TMB), which were frequently used substrates for testing peroxidase activity. Moreover, Au clusters had a precise structural formula and contains 26 gold atoms. The constructed Au clusters had the following functions: (1) specifically binding to MT1-MMP by the sequence of peptides; (2) marking the transmembrane protein MT1-MMP on cells with a red fluorescent label by virtue of the inherent fluorescent properties of Au clusters; (3) catalytic color imaging of MT1-MMP on the cell membrane in the presence of DAB; (4) precisely quantifying the expression level of MT1-MMP by ICP-MS. In this work, the human cervical cancer Siha, Caski and Hela cells with different risks of invasion/metastasis were selected as models. As expected, the constructed Au clusters could specifically label MT1-MMP on the surface of cervical cancer cells. In addition, it could distinguish the expression levels of MT1-MMP on Siha, Caski and Hela cells through fluorescence imaging and DAB color imaging. More importantly, the clusters-labeled cervical cancer cells were quantitatively distinguished by counting Au using ICP-MS (Scheme 1). This protein detection and analysis method that used a single metal clusters probe to achieve multifunctional integration had great application prospects in the analysis of disease-related proteins.

## 2. Results and Discussion

### 2.1. Preparation and Characterization of the Au Clusters

**Figure 1:**
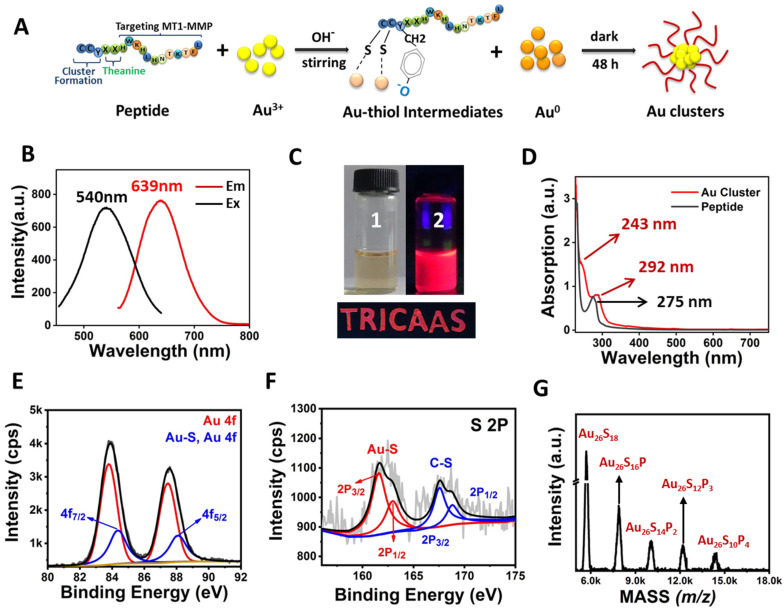
Characterization of Au_26_ cluster. (**A**) The formation of Au clusters. (**B**) Fluorescence excitation and emission spectra of Au clusters. (**C**) Photo images of Au clusters under visible (1) and a portable UV lamp (2). The excellent fluorescence properties of Au clusters can make them be used as fluorescent inks. e.g., “TRICAAS” was written using Au clusters emitting red fluorescence under a portable UV lamp. “TRICAAS” is the abbreviation of our affiliation institution. (**D**) Absorption spectra of peptide and Au clusters. (**E**,**F**) XPS spectrum of Au 4f and S 2P of Au clusters. (**G**) MALDI-TOF-MS spectrum of Au clusters. The mass of the Au_26_P_9_ molecule fragment could be written by Au_26_P_x_S_18–2x_, where x ranges from 0 to 9, P represents the peptide, and S represents sulfur.

The preparation method of Au clusters was similar to the method we previously reported [39,40]. The peptide (H_2_N-CCY-XX-HWKHLHNTKTFL-COOH) contains three domains. Domain 1 was CCY, the sulfhydryl of cysteine (C) and phenolic groups of tyrosine (Y) could capture the Au cluster via the Au–S bond. Domain 2 (XX) was two theanine, which was a functional secondary metabolite in tea plants, to enhance the biocompatibility and separate the Domain 1 and Domain 2 of peptide. Domain 3 was the sequence (HWKHLHNTKTFL) for binding to MT1-MMP protein [41]. A schematic diagram of the construction of Au clusters is depicted in Figure 1A.

**Figure 2:**
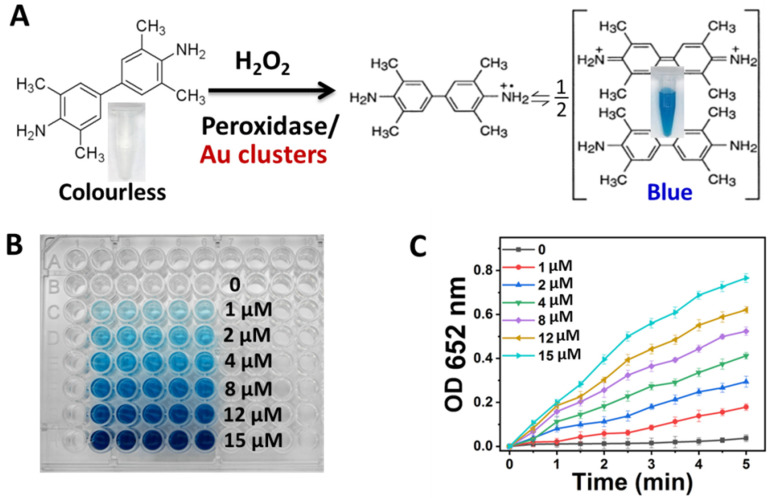
Peroxidase activity of Au clusters. (**A**) Structural changes of TMB after catalytic oxidation. (**B**) The color changes of TMB (1 mM) mixed with H_2_O_2_ (100 mM) and different concentrations of Au clusters. (**C**) TMB absorbance value change at 652 nm after mixture with H_2_O_2_ (100 mM) and different concentrations of Au clusters.

The as-synthesized Au clusters were purified using differential centrifugation and ultrafiltration tube molecular sieves. After purification, the fluorescence and absorption spectra of the Au clusters were measured. As shown in Figure 1B, Au clusters exhibited an intense emission peak located at 639 nm and an excitation peak at 540 nm. The synthesized Au clusters were uniform and transparent solutions under natural light but exhibited red fluorescence under UV irradiation of 365 nm (Figure 1C). The fluorescent properties of Au clusters could make them used as fluorescent inks (Figure 1C). As depicted in Figure 1D, the absorption peak position of peptide is located at 275 nm, while the position of the absorption peak of Au clusters is located at 243 and 292 nm, which was the characteristic absorption peaks of peptide-Au clusters [42,43]. As shown in Figure S1, the peaks of C, Au, S, O, and N were obviously detected by X-ray photoelectron spectroscopy (XPS) spectrum. Two intense peaks at 84.35 and 88.08 eV were assigned to the binding energy of Au 4f_7/2_ and Au 4f_5/2_ (Figure 1E). This was consistent with the XPS results of thiol-protected Au clusters [28,44]. Meanwhile, the peak position of binding energy at 161.63 and 163.04 eV were S 2p_3/2_ and S 2p_1/2_, respectively [Figure 1F]. These data further verified the conjugation between Au and the sulfhydryl group of cysteine. Moreover, matrix-assisted laser desorption/ionization time-of-flight mass spectrometry (MALDI-TOF MS) was used to obtain the cluster’s structural composition [45,46,47]. After being purified and collected using protein purification systems, the sample was detected by MALDI-TOF MS. As shown in Figure 1G, a series of mass spectrum peaks between 5.5 K and 18.0 KDa were clearly visible. As the C-S bond of the peptide could be easily broken under MS laser radiation, peptides could be stripped from the Au core, but their S atoms were left to bind the Au atom [45,48]. As there were two S atoms in one peptide, the distance among adjacent mass spectra peaks is consistent with the m/z of the loss of two S atoms in a peptide (Figure 1G). The most abundant mass spectrum peak at 5698 Da could be expressed by Au_26_S_18_, e.g., the molecular formula of one Au cluster was Au_26_P_9_. High-resolution transmission electron microscopy (HRTEM) images demonstrated that Au clusters were ultra-small nanoparticles (Figure S2). The average zeta potential of Au clusters was −31.57 ± 0.92 mV, indicating their good stability in physiological solutions (Figure S3). The fluorescence intensity of Au clusters was highly stable under the different solutions of H_2_O, PBS, medium and with no obvious decrease within 90 days (Figure S4). Conversely, the constructed Au clusters exhibited peroxidase-like properties. TMB is a colorless and transparent solution and could be oxidized to blue with a maximum absorbance at 652 nm in the presence of catalase and H_2_O_2_ (Figure 2A) [49,50]. As depicted in Figure 2B, with the concentration of Au clusters increased, the color of the mixture samples became blue and gradually darkened. The value of maximum absorption peak position at 652 nm of samples was also recorded to quantify the color change of TMB after Au clusters treatment. As the concentration of Au clusters in the system increased within 5 min, the absorbance value also increased (Figure 2C). As expected, there was no significant change in the absorbance value of TMB without Au clusters treatment (Figure 2B,C). These results indicated that Au clusters had good peroxidase-like properties.

### 2.2. MT1-MMP Expression Levels of the Human Cervical Cancer Cells with Different Invasion/Metastasis

**Figure 3:**
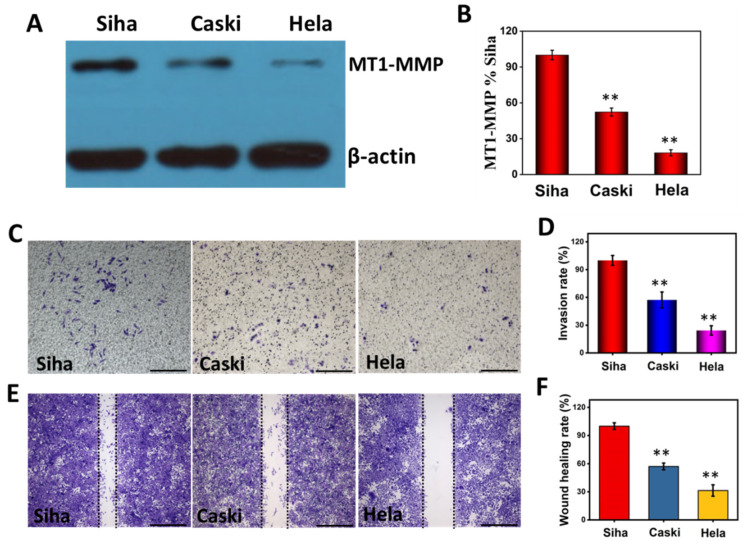
(**A,B**) The expression level and analysis of MT1-MMP in Siha, Caski and Hela cells. (**C,D**) Photo images and analysis of invasion ability of Siha, Caski and Hela cells after 24 h incubation. (**E,F**) Photo images and analysis of migration ability of Siha, Caski and Hela cells after 24 h incubation. Scale bars, 200 μm. ** *p* < 0.01.

To verify the relationship between cell invasiveness and MT1-MMP expression, we first detected the expression level of MT1-MMP in human cervical carcinoma Siha, Caski and Hela cells using a commercialized immunoblotting method. As shown in Figure 3A, cervical carcinoma Siha cell populations expressed more MT1-MMP than Caski and Hela cell populations. The expression level of MT1-MMP on Caski was between Siha and Hela cells. These three protein bands were analyzed using ImageJ software. As shown in Figure 3B, the amount of MT1-MMP in Caski and Hela cell populations was 52.29% and 18.12% of that in Siha cell populations, respectively.

The invasion/metastasis of Siha, Caski and Hela cells were assessed with invasion and wound healing assays, respectively. The transwell chamber was coated with Matrigel. Cancer cells with higher invasion ability will degrade the Matrigel coating and grow on the back of the chamber [51,52]. As depicted in Figure 3C, compared with Caski and Hela cells, the greatest number of Siha cells through the Matrigel showed the strongest invasion ability. If the invasion rate of Siha was defined as 100%, the invasion rate of Caski and Hela cells were 57.30% and 24.34% of that Siha cells, respectively (Figure 3D). Moreover, wound healing assays were performed to assess the migration ability of Siha, Caski and Hela cells. Compared with Caski and Hela cells, Siha cells demonstrated the strongest migration ability after the creation of single cell layer wound (Figure 3E). If the wound healing rate of Siha was 100%, the wound healing rate of Caski and Hela cells were 57.14% and 31.42%, respectively (Figure 3F). These results demonstrated that the invasion/migration abilities of Siha cells were higher than those of Caski and Hela cell lines. The invasion/migration abilities of Siha, Caski and Hela cell populations were consistent with their MT1-MMP expression levels (Figure 3A,B). Our results suggested that the amount of MT1-MMP on cells was directly related to the invasion/metastasis abilities of cervical cancer cells.

### 2.3. Specific Marking of MT1-MMP on Cervical Cancer Cells

**Figure 4:**
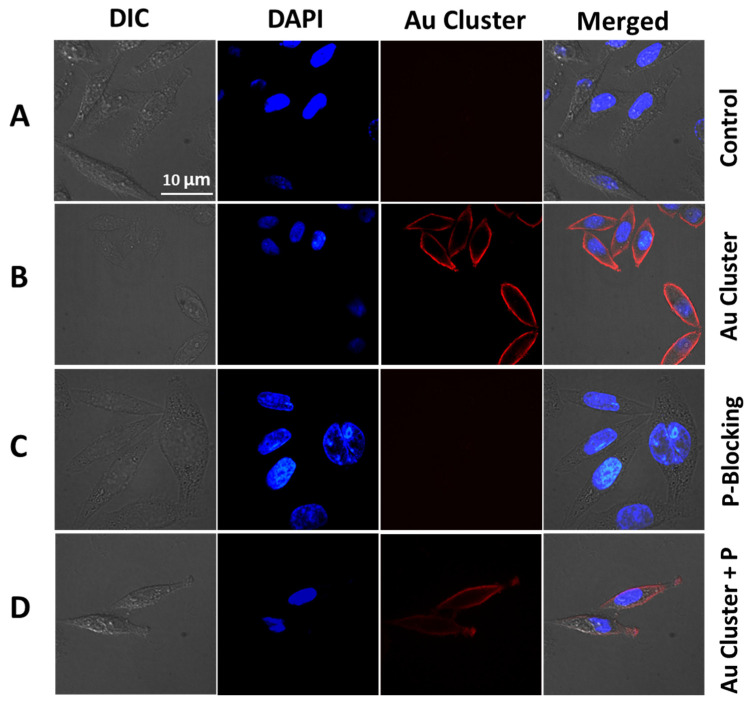
Au clusters specifically target MT1-MMP in cervical cancer Siha cells. (**A**) CLSM images of Siha cells treated with PBS. (**B**) Siha cells are marked by Au clusters (8 µM for 30 min). (**C**) Siha cells were first treated with free peptides (2 mM for 1 h), followed by the addition of Au clusters (8 µM for 30 min). (**D**) Free peptides (100 µM) and Au clusters (8 µM) were introduced to Siha cells at the same time and incubated for 30 min. The blue fluorescence is the DAPI for the cell nuclei.

Confocal laser scanning microscopy (CLSM) (PerkinElmer, Waltham, MA, USA) was used to visually see the specific targeting of MT1-MMP in cervical cancer Siha cells. After incubating with Au clusters, the cell surface showed bright red fluorescence, while the control group had no fluorescence signal (Figure 4A,B). As mentioned above, the targeting of Au clusters came from the functional peptide sequence on its surface. To prove the specific binding of Au clusters and MT1-MMP, a blocking experiment was performed. As shown in Figure 4C, Siha cells were first co-cultivation with free peptide. Then, Au clusters were added to Siha cells and incubated for another 30 min. Since all the MT1-MMP binding sites were occupied by peptide, there was no fluorescence on Siha cells (Figure 4C). Another study was performed by adding the Au clusters and peptide to Siha cells simultaneously. As expected, compared with the only Au clusters treatment, there was only faint fluorescence on Siha cells, suggesting Au clusters and peptide competed for the same binding site (Figure 4D). These results collectively suggested that Au_26_ cluster could specifically bind to MT1-MMP on Siha cells (Figure 4).

### 2.4. Optimized Labeling Experiment of MT1-MMP by Au Clusters on Siha Cells

**Figure 5:**
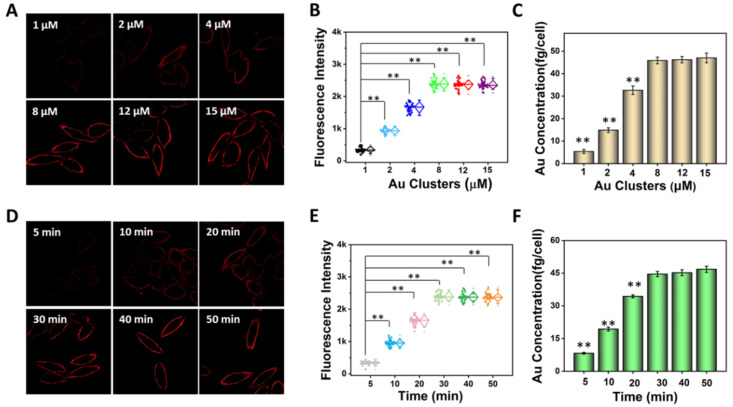
Optimization of labeling conditions of Au_26_ cluster for MT1-MMP. (**A**) Fluorescence imaging of Siha cells treated with different concentrations of Au clusters for 30 min. (**D**) Fluorescence imaging of Siha cells treated with 8 μM Au clusters at different times. (**B**,**E**) Fluorescence intensity analysis corresponds to A and D. Each point represents the fluorescence intensity of a cell. (**C**,**F**) The average amount of Au determined by ICP-MS on Siha cells corresponding to A and D. ** *p* < 0.01.

To accurately observe the MT1-MMP on the cervical cancer cell membrane, an optimized incubation condition for the labeling of Siha cells with Au clusters was performed. Au clusters with different concentrations (1, 2, 4, 8, 12, and 15 µM) were introduced into Siha cells for 30 min at 37 °C. When the concentration of Au clusters at 1 μM, the fluorescence intensity on Siha cells was weak (Figure 5A). The fluorescence intensity of Siha cells increased with the increase of the concentration of Au clusters from 1 to 8 μM (Figure 5A). At the concentration of 8 μM, the fluorescence intensity of the clusters-labeled cells was saturated, and no longer obvious changes at higher concentrations (12 and 15 μM), indicating that all MT1-MMP sites on Siha cells were bound by Au clusters (Figure 5A,B). On the other hand, the optimal marking conditions were confirmed by ICP-MS. Briefly, after fluorescence imaging, a large number of Siha cell populations were digested with aqua regia to quantify the amount of Au, and the cells were counted by flow cytometry. The average Au mass on each clusters-labeled Siha cell was calculated based on the standard curve (Figure S5). As shown in Figure 5C, the content of Au on each cell increased with the increase of the concentration of Au clusters and remained unchanged after the concentration of 8 μM Au clusters treatment. This result was consistent with the results of fluorescence imaging (Figure 5A,B). Clusters-labeling tests at different time points were performed to determine the optimum labeling time. As depicted in Figure 5D, Siha cells were treated with 8 μM Au clusters with different time periods. As the co-incubation time increased from 5 to 50 min, the fluorescence intensity of Siha cells increased and there were no significant changes at longer incubation times (Figure 5D,E). Moreover, the quantitative results of ICP-MS also showed that the Au content on the labeled-cells reached saturation at the labeling time of 30 min (Figure 5F). These results collectively confirmed that the optimal Siha cell labeling condition was 30 min when Au clusters at 8 μM.

### 2.5. Quantitatively Distinguish Cervical Cancer Cells with Different Invasion/Metastasis Capabilities

**Figure 6:**
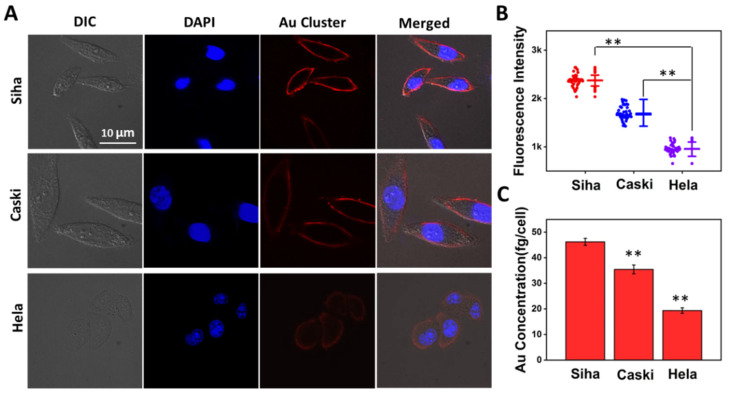
Quantitatively distinguish cervical cancer cells with different invasion/metastasis capabilities. (**A**) Human cervical cancer Siha, Caski and Hela cells were exposed to 8 μM Au clusters for 0.5 h. (**B**) Fluorescence intensity analysis corresponds to A. Data on the left cell line represent the fluorescence intensity for each of the 50 cells selected, and data on the right represent the average fluorescence intensity and standard deviation for the 50 cells, respectively. (**C**) The average Au mass on each cell was quantified by ICP-MS. ** *p* < 0.01.

We subsequently demonstrated whether the Au clusters could quantitatively distinguish cervical cancer cells with different invasion/metastasis capabilities. As shown in Figure 6A, the labeled-cells were imaged using a fluorescence microscope after treatment with Au clusters (8 μM, 30 min). The red fluorescence on the Siha cells membrane was the brightest, followed by Caski cells, and the Hela cells only emitted weak fluorescence (Figure 6A). We randomly selected 50 cells for fluorescence intensity statistics and found that the fluorescence intensity on Siha cells was the strongest than that on Caski and Hela cells (Figure 6B). After using CLSM for fluorescence qualitative differentiation, ICP-MS was used to quantify the Au content on clusters-labeled cervical cancer cells. As shown in Figure 6C, the average Au counts on Siha, Caski, and Hela cells were 46.21 ± 1.37, 35.42 ± 1.72, 19.34 ± 1.09 fg/cell, respectively. The Au content in Siha cells was the most, which was consistent with the fluorescence intensity (Figure 6A,B). In other words, compared with Caski and Hela cells, Siha cells expressed the most MT1-MMP and had the strongest ability to invade and metastasize. This fluorescence imaging and ICP-MS quantitative results matched the commercial Western Blotting results data in Figure 3A,B. However, Western Blotting required processing a large number of cell populations, and part of the protein sample was lost during the process of cell disruption and protein extraction.

**Figure 7:**
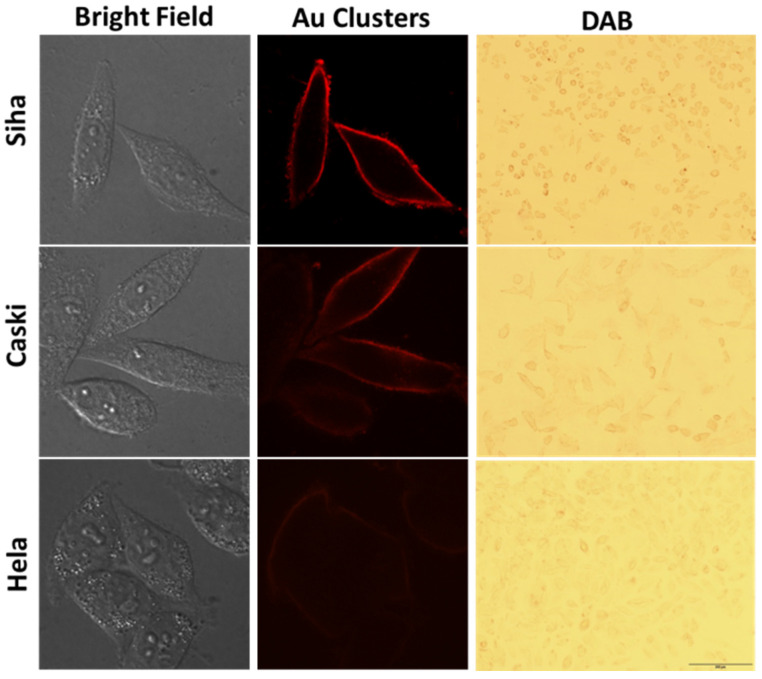
Quantitatively distinguish cervical cancer cells with different invasion/metastasis capabilities. The first column on the left is the bright field images, the middle column is the representative images of cells labeled with Au clusters (8 µM for 30 min), the third column is the representative images of the clusters labelled-cells after DAB color imaging.

On the other hand, we could quickly qualitatively distinguish cervical cancer cells with different invasion/metastasis capabilities using Au clusters’ peroxidase-like properties. After labeled-cells were fluorescence imaged by the CLSM system, DAB solution was introduced into the samples and imaged via an optical microscope. The Au clusters bound by MT1-MMP on the cell surface could catalyze DAB, where a brown precipitate formed on the cell membrane. As shown in Figure 7, among the three types of cells, the brown precipitate on Siha cells surface was the darkest. There was no obvious brown color attached to the Hela cells, suggesting that Hela cells had the least MT1-MMP among the three types of cervical cancer cells (Figure 7). These results clearly demonstrated that the Au_26_ cluster could distinguish the amount of MT1-MMP on cervical carcinoma Siha, Caski and Hela cells, that is, distinguish the invasion/metastasis capabilities of the three types of cells (Figure 3, Figure 6 and Figure 7).

## 3. Conclusions

MT1-MMP has been used as a biomarker for tumor diagnosis and prognosis of multiple types of cancers. The expression level of MT1-MMP was directly related to the invasion/metastasis ability of cancer cells. Here, we established a simple and accurate analysis method to directly and quickly distinguish MT1-MMP expression levels in cervical cancer cells. In summary, we used an artificially designed peptide to construct a multifunctional Au_26_ cluster probe with fluorescence and peroxidase properties. The constructed Au clusters exhibited good stability and could specifically recognition MT1-MMP in human cervical cancer cells. The MT1-MMP level of Siha, Caski and Hela cells could be distinguished by CLSM fluorescence imaging and precisely ICP-MS quantification. Furthermore, Au clusters attached to cervical cancer cells could catalyze DAB in the presence of H_2_O_2_, giving a brown label to MT1-MMP on cell surface. The MT1-MMP expression of three cervical cancer cells could be distinguished by visual observation of the brown color on cell membrane without the aid of equipment. With further development and optimization, multifunctional integrated Au clusters may have great potential to quickly distinguish the invasion/metastasis ability of clinical tumor cells.

## 4. Materials and Methods

### 4.1. Materials

The peptide (H_2_N-CCYXXHWKHLHNTKTFL-COOH) was synthesized using commercial solid-phase synthesis methods. Nitric acid (HNO_3_), Sodium hydroxide (NaOH), Hydrogen tetrachloroaurate (III) (HAuCl_4_·3H_2_O), hydrogen peroxide (H_2_O_2_) and hydrochloric acid (HCl) were purchased from Sinopharm Chemical Reagent Co., Ltd. Siha, Caski and Hela cell lines were obtained from ATCC. The RPMI 1640 cell culture medium, DMEM High Glucose and PBS were acquired from HyClone, Logan, UT, USA. FBS and trypsin-EDTA were purchased from Gibco, CA, USA. 3, 3′ 5, 5′-tetramethyl benzidine sulfate (TMB), DAB HRP Color Development Kit, DAPI staining solution, anti-rabbit IgG-HRP, Cell lysis buffer, a BCA Protein Assay Kit and other cell molecular biology reagents were purchased from the Beyotime Institute of Biotechnology, Shanghai, China. Human MT1-MMP antibody and anti-rabbit secondary antibodies were purchased from Abcam Biotechnology. Ultrapure water (18 MΩ) was used throughout the study.

### 4.2. Synthesis of Au clusters

Briefly, a mass of 5 mg peptide was dissolved in a volume of 1300 μL aqueous solution. Then, a volume of 100 μL HAuCl_4_ (25 mM) was dripped into the sample under vigorous stirring (1000 rpm) at room temperature (25 °C). Next, the pH of the reaction system was adjusted to alkalinity (about pH 10) by NaOH aqueous solution. Last, the sample was sealed and stored in the dark for 48 h.

### 4.3. Purification and Quantification of Au Clusters

The as-synthesized Au clusters were purified by using high-speed centrifugation, molecular sieve (Millipore) and protein purification system to remove the formed nanoparticles, free peptides and gold ions. First, Au clusters were centrifuged (10,000× *g* for 20 min) to remove nanoparticles. Next, the sample was separated by a molecular sieve (Millipore, MWCO: 30K) with a centrifugal force of 7000× *g*. Then, the sample of the outer tube aqueous solution was collected. Next, the sample was transferred into the Ultracel-10K centrifugal filter to remove ion and free peptide at a centrifugal force of 7000× *g*. Then, the sample was centrifuged and ultrafiltered by adding buffer until the outer tube liquid had no absorption peak at 275 nm which was the absorption peak of tyrosine on the peptide. Last, Au clusters in the inner tube were acquired and further purified The Au clusters was purified using protein purification systems (GE AKTA PURE). The chromatographic column is GE Healthcare Columu Superdex 200 10/300 GL. The components of the mobile phase are 0.5 M arginine and 1 M NaCl solution [43]. Au clusters was collected under a UV detector. Finally, Au clusters were stored away from light.

ICP-MS analysis systems were employed to acquire the concentration of Au clusters. After purification, 5 μL Au clusters were injected into aqua regia for digestion overnight. Then, the mixed samples were evaporated in a BHW-09C Heating Block system. Next, samples were diluted by the mixed acid of 2% HNO_3_ and 1% HCl solution. The 20 ppb bismuth in a mixed acid solution was used as an internal standard. A series of Au standard solutions (0.5, 1, 5, 10, 25 and 50 ng/mL) was used to draw an Au standard curve. Last, all samples were quantified by ICP-MS. The product yield of the Au clusters was 53.28%.

### 4.4. Characterization of the Au Clusters

The fluorescence spectrum was obtained using a spectrofluorometer (F-7000, Hitachi, Tokyo, Japan). The ultraviolet–visible spectrum of Au clusters was measured by a UV-1800 spectrophotometer. The molecular composition of the Au cluster was identified by matrix-assisted laser desorption/ionization time-of-flight mass spectrometry (MALDI-TOF MS). The operating mode is an ABI MALDI-TOF system in positive ion linear mode, and α-cyano-4-hydroxycinnamic acid was used as the matrix. The XPS spectrum and zeta potential were acquired by an X-ray photoelectron spectrometer (ESCALAB 250Xi, Thermo, Waltham, MA, USA) and zeta potential analyzer (90Plus Zeta, NanoBrook, New York, NY, USA), respectively.

### 4.5. Peroxidase-like Activity of Au Clusters

The catalytic ability of Au clusters was detected by oxidizing TMB in the presence of H_2_O_2_. In the catalysis of horseradish peroxidase, the colorless TMB can become a blue water-dissolved product. This product has a maximum absorption peak at 652 nm. The catalytic ability of Au clusters was tested by mixing H_2_O_2_, TMB and Au clusters. This mixed solution system contained 100 mM H_2_O_2_, 1 mM TMB and different concentrations of Au clusters (0, 1, 2, 4, 8, 12, and 15 µM). These catalytic reactions were performed in a 96-well plate. Finally, the absorption values (λ_652nm_) at different reaction time points were monitored by a microplate reader (SpectraMax M2).

### 4.6. Specificity Study of Au Clusters to MT1-MMP

Briefly, after being fixed with 3.7% paraformaldehyde for 30 min, Siha cells were washed with PBS three times. Then, cells were treated with 8 µM Au clusters for 0.5 h at 37 °C. The Siha cells in the control group were treated with PBS. The cell nuclei were stained with DAPI (5 µg/mL, 5 min). Finally, the Siha cells were imaged by UltraVIEW Vox (PerkinElmer, Waltham, MA, USA) CLSM attachment and a Nikon Ti-e microscope with a 60 × 1.4 numerical aperture plan apochromatic oil immersion lens.

### 4.7. MT1-MMP Binding Site Blocking Experiment

A blocking experiment was also performed to verify the specificity of Au clusters recognition MT1-MMP. Siha cells were fixed with 3.7% paraformaldehyde for 30 min. Then, free peptide solution (2 mM) was added to cells for 1 h. Next, a volume of 2 mL Au clusters solution (8 µM) was introduced into cells for 30 min. Then, the nuclei of cells were stained with DAPI (5 µg/mL) for 5 min. Finally, the cells were imaged by the fluorescence microscope.

### 4.8. Competitive Targeting Study of Au Clusters and Peptide

Siha cells were fixed with 3.7% paraformaldehyde for 30 min. After washing with PBS, a volume of 2 mL mixed solution (peptide, 100 µM and Au clusters, 8 µM) was added into Siha cells for 0.5 h. Next, the nuclei of cells were stained with DAPI (5 µg/mL) for 5 min before being observed using a fluorescence microscope.

### 4.9. Quantitative Analysis of MT1-MMP on Cells via ICP-MS

The expression of MT1-MMP protein on the cell was quantified by the ICP-MS analysis system. After cells and Au clusters (8 µM) were co-incubated together for 30 min, the cells were transferred into a MARS vessel for digestion overnight using 3 mL aqua regia. Then, all acid in the sample was evaporated in a BHW-09C Heating Block system. After that, the samples were diluted using the mixed acid of 2% HNO_3_ and 1% HCl solution. Next, all the samples were detected using the ICP-MS. A portion of the cells drawn from each sample was counted by flow cytometry. The experiment was repeated three times.

### 4.10. The Amount of MT1-MMP on Siha, Caski and Hela cells Was Evaluated by Commercial Immunoblotting

First, Siha, Caski and Hela cells (1 × 10^7^) were washed with PBS three times. Then, cells were lysed with lysis buffer (Tris-HCl (pH 7.5), 1 mM Na_2_EDTA, 2.5 mM sodium pyrophosphate, 1 mM PMSF, 1 μg/mL leupeptin, 1 mM β-glycerophosphate, 1 mM orthovanadate, 1% Triton, 1 mM EGTA, and 150 mM NaCl). After that, cell membrane protein was extracted by a Membrane Proteins Extraction Kit (Beyotime, Shanghai, China). The concentration of samples was quantified by a BCA assay before loading into the gel plate. Next, the extracting protein samples were mixed with 1x loading buffer. The same amount of protein was loaded and separated by sodium dodecyl sulfate-polyacrylamide gel electrophoresis (SDS-PAGE). A rabbit MT1-MMP antibody was incubated with the PVDF membranes overnight at 4 °C. The next day, a secondary antibody was used to incubate with them for 2 h with shaking. The gray value of protein bands was quantitatively calculated using Image J software.

### 4.11. Wound Healing Assay

Siha, Caski and Hela cells were seeded into 6-well plates. A straight line was scratched with a pointed tweezers when the cells form a monolayer on the bottom of the culture plates. Then, cells were washed with PBS three times to remove damaged cells. A volume of 2 mL serum-free medium was added into cells for 24 h at 37 °C. After that, cells were fixed with 3.7% paraformaldehyde in PBS solution for 30 min and washed with PBS three times before being stained with 0.1% crystal violet for 8 min. Then, all the samples were imaged under a microscope and the wound width was recorded for each sample. The migration distance of Siha was defined as 100% to calculate the migration ability of other cells.

### 4.12. Transwell Invasion Assay

The insert membrane of the Transwell plate was coated with COL-I (100 µL, 300 mg/mL). Then, the plate was dried at 37 °C overnight. The next day, the plate with COL-I coating was soaked in serum-free medium for 1 h. Then, 100 μL of Siha, Caski and Hela cells (2 × 10^5^ cells/mL in serum-free medium) was added into the chamber coated with COL-I in the Transwell plate. The 10% FBS medium was added into the outer chamber of the plate as a chemoattractant. Then, the plate was incubated for 24 h at 37 °C. Next, the COL-I coating was removed and cells on the lower surface of the membrane were fixed with 3.7% paraformaldehyde. Then, cells were washed with PBS and stained with 0.1% crystal violet for 10 min. Finally, cells were imaged with a microscope and counted in different fields of view. The number of invasive Siha cells was defined as 100% to calculate the invasive ability of other cells.

### 4.13. Statistical Analysis

All quantitative data analyses were reported as mean ± s.d. Statistical analysis was assessed by the one-way analysis of variance (ANOVA). The significant differences between groups were expressed by * *p* < 0.05, ** *p* < 0.01.

## Data Availability

The data are available on reasonable request from the corresponding author.

## Supplementary Materials

The following supporting information can be downloaded at: https://www.mdpi.com/article/10.3390/nano12010145/s1, Figure S1: The survey XPS spectrum of Au clusters; Figure S2: HRTEM image of Au clusters; Figure S3: The zeta potential of Au clusters. “Au cluster-1,2,3” means that we have repeatedly measured the Zeta Potential of the Au clusters three times; Figure S4: The stability of Au clusters in the system of H_2_O, PBS, medium and stored at room temperature for 90 days; Figure S5. Calibration curve of Au standards by ICP-MS.
