# Supplementary material for: Qualitative and Quantitative Analysis of Tumor Cell Invasion Using Au Clusters
Source: Nanomaterials (Basel). 2021 Dec 31;12(1):145. doi: 10.3390/nano12010145 (PMC8746878; doi:10.3390/nano12010145)
Supplement: Supplementary file 1 [file nanomaterials-12-00145-s001.zip › nanomaterials-1482060-supplementary.pdf]

Supporting Information

Qualitative and Quantitative Analysis of Tumor Cell Invasion Using Au Clusters

Xiangchun Zhang [1,†], Qinqin Zheng [1,†], Ziqi Wang [1], Chao Xu [2], Haolei Han [1], Aiping Li [1], Guicen Ma [1], Jiaojiao Li [3], Chengyin Lu [1], Hongping Chen [1,*] and Zhichao Zhang [4,*]

[1] Tea Research Institute, Chinese Academy of Agricultural Sciences, Hangzhou 310008, China; zhangxc@tricaas.com (X.Z.); zhengqinqin@tricaas.com (Q.Z.); wangziqi199710@163.com (Z.W.); 18482004388@163.com (H.H.); AdelapLive@outlook.com (A.L.); mgc1314@tricaas.com (G.M.); lchy@tricaas.com (C.L.)
[2] College of Chemistry and Material Science, Shandong Agricultural University, Taian 271018, China; xuc@sdau.edu.cn
[3] Department of Chemistry and Biology, Faculty of Environment and Life Science, Beijing University of Technology, Beijing 100124, China; lijiaojiao@emails.bjut.edu.cn
[4] Department of Musculoskeletal Tumor, Fudan University Shanghai Cancer Center, Shanghai 200032, China
* Correspondence: thean27@tricaas.com (H.C.); zhichao90@163.com (Z.Z.)
† These authors contributed equally to this work.

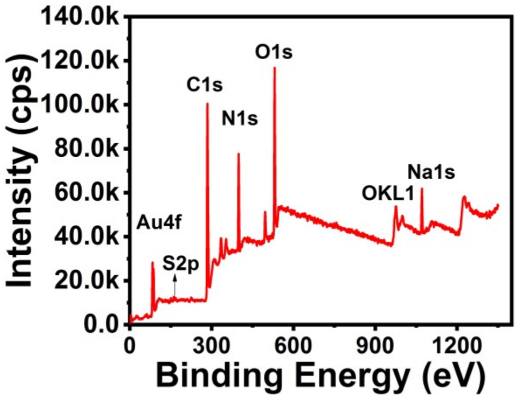

Figure S1. The survey XPS spectrum of Au clusters.

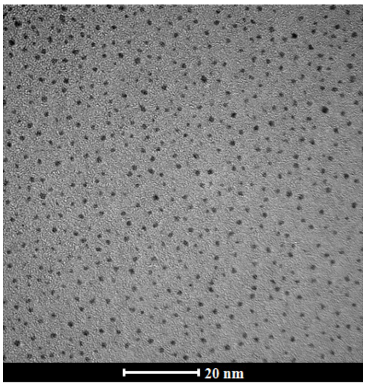

Figure S2. HRTEM image of Au clusters.

Sample ID	Zeta Potential (mV)
Au clusters-1	-31.58
Au clusters-2	-30.64
Au clusters-3	-32.49
Mean:	-31.57

Figure S3. The zeta potential of Au clusters. "Au cluster-1,2,3" means that we have repeatedly measured the Zeta Potential of the Au clusters three times.

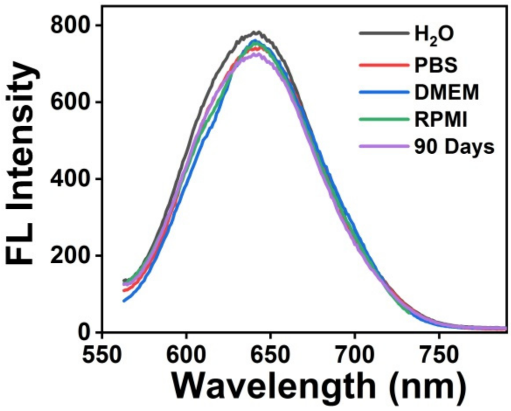

Figure S4. The stability of Au clusters in the system of H_2O, PBS, medium and stored at room temperature for 90 days.

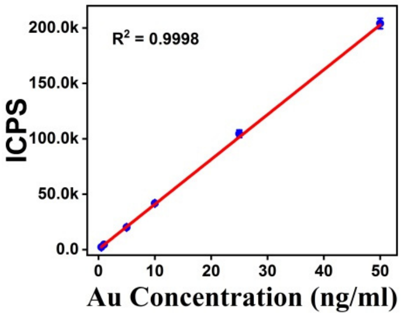

Figure S5. Calibration curve of Au standards by ICP-MS.
